# Nornidulin, A New Inhibitor of *Plasmodium falciparum* Malate: Quinone Oxidoreductase (*Pf*MQO) from Indonesian *Aspergillus* sp. BioMCC f.T.8501

**DOI:** 10.3390/ph16020268

**Published:** 2023-02-10

**Authors:** Alfian Wika Cahyono, Loeki Enggar Fitri, Sri Winarsih, Erwahyuni Endang Prabandari, Danang Waluyo, Amila Pramisandi, Evita Chrisnayanti, Diana Dewi, Eka Siska, Nurlaila Nurlaila, Nuki Bambang Nugroho, Tomoyoshi Nozaki, Suciati Suciati

**Affiliations:** 1 Doctoral Program in Medical Science, Faculty of Medicine, Universitas Brawijaya, Malang 65145, East Java, Indonesia; 2 Department of Parasitology, Faculty of Medicine, Universitas Brawijaya, Malang 65145, East Java, Indonesia; 3 Malaria Research Group, Faculty of Medicine, Universitas Brawijaya, Malang 65145, East Java, Indonesia; 4 Department of Microbiology—Department of Pharmacy, Faculty of Medicine, Universitas Brawijaya, Malang 65145, East Java, Indonesia; 5 Research Centre for Vaccine and Drug, National Research and Innovation Agency, Cibinong Science Centre, Jalan Raya Bogor, Bogor 16143, West Java, Indonesia; 6 Research Centre for Applied Microbiology, National Research and Innovation Agency, Cibinong Science Centre, Jalan Raya Bogor, Bogor 16143, West Java, Indonesia; 7 Department of Biomedical Chemistry, Graduate School of Medicine, The University of Tokyo, Bunkyo-ku, Tokyo 113-8654, Japan; 8 Department of Pharmaceutical Science, Faculty of Pharmacy, Universitas Airlangga, Surabaya 60115, East Java, Indonesia

**Keywords:** *Aspergillus* sp. BioMCC f.T.8501, malaria, nornidulin, *Pf*MQO, *Plasmodium falciparum*, purification

## Abstract

This study aimed to obtain a microbial active compound as a novel antimalarial drug from Indonesian isolates. Target-based assays were used to screen for antimalarial activity against the parasite mitochondrial, *Plasmodium falciparum* malate:quinone oxidoreductase (*Pf*MQO) enzyme. In total, 1600 crude extracts, composed from 800 fungi and 800 actinomycetes extracts, were screened against *Pf*MQO, yielding six active extracts as primary hits. After several stages of stability tests, one extract produced by *Aspergillus* sp. BioMCC f.T.8501 demonstrated stable *Pf*MQO inhibitory activity. Several purification stages, including OCC, TLC, and HPLC, were performed to obtain bioactive compounds from this active extract. All purification steps were followed by an assay against *Pf*MQO. We identified the active compound as nornidulin based on its LC-MS and UV spectrum data. Nornidulin inhibited *Pf*MQO activity at IC_50_ of 51 µM and *P. falciparum* 3D7 proliferation in vitro at IC_50_ of 44.6 µM, however, it had no effect on the growth of several mammalian cells. In conclusion, we isolated nornidulin from Indonesian *Aspergillus* sp. BioMCC f.T.8501 as a novel inhibitor of *Pf*MQO, which showed inhibitory activity against the proliferation of *P. falciparum* 3D7 in vitro.

## 1. Introduction

Malaria is an infectious disease caused by a parasite of the genus *Plasmodium,* which is transmitted through the bite of a female *Anopheles* mosquito. The World Health Organization reported that there were 241 million cases of malaria in 2020, with a mortality rate of 627,000, a 12% increase over 2019 [1]. One of the *Plasmodium* species causing severe and deadly malaria in humans is *Plasmodium falciparum* [2]. To date, malaria is treated by first-line chemotherapeutic agents, including artemisinin, artemether, artesunate, arteether, and its derivative, dihydroartemisinin. However, the single use of these drugs has been reported to induce drug-resistance parasites; therefore, the WHO recommended artemisinin-based combination therapy (ACT) for effective treatment and to reduce the incidence of malaria [1,3]. Unfortunately, the efficacy of ACT has recently been reported to be declining. Evidence of resistance to malaria treatment in Cambodia and the Great Mekong sub region in Southeast Asia have been shown by the delayed clearance of parasites following ACT treatment of falciparum malaria [4,5]. Therefore, artemisinin-based triple combination therapy (TAC) was initiated for better treatment outcomes [5,6]. As the efficacy of current antimalarial drugs has been threatened by parasite resistance, efforts to discover new antimalarial agents are urgently needed [7,8].

Currently, ongoing efforts in the development of antimalarial agents exhibiting novel mechanisms of action are deemed important [3]. Several drug discovery strategies, including high throughput screening (HTS), have been used to identify active compounds against *Plasmodium* cells (whole-cell approach). Another breakthrough is the use of *Plasmodium* biochemical target proteins (target-based approach) [9]. Recently, enzymes involved in the mitochondrial electron transport chain (mtETC) gained attention as potential targets for anti-malarial drug development. *Plasmodium* mtETC contains five dehydrogenases: type II NADH dehydrogenase (NDH2), malate quinone oxidoreductase (MQO), dihydroorotate dehydrogenase (DHODH), glycerol 3-phosphate dehydrogenase (G3PDH), and succinate dehydrogenase (SDH) [10]. *Plasmodium falciparum* malate:quinone oxidoreductase (*Pf*MQO) is reported to exhibit promising targets in the development of new antimalarial drugs [8]. In addition to its roles in the mtETC, tricarboxylic acid, and fumarate cycles, *Pf*MQO is also critical for the *P. falciparum* life cycle at the asexual (erythrocytic) stage. Because MQO is not present in humans, this specific target reduces the risk of adverse effects in humans [8,11,12].

On the other side, microbes have become an important source for antimalarial drug discovery. *Streptomyces* sp. BioMCC-a.EP.1039 was found to produce borrelidin, a potent agent that inhibits the proliferation of *P. falciparum* 3D7 strains, as well as chloroquine/pyrimethamine/sulfadoxine-resistant K1 strains both in vitro and in vivo [12,13]. Several secondary metabolites derived from the endophytic fungus *Aspergillus unguis* BCC54176 have recently been reported to have a wide range of antimicrobial activities, including antimalarial activity, against *Plasmodium falciparum* (K1, multidrug-resistant strain) [14]. Despite the potential of microbes as a source for anti-malarial drug discovery, there is no report that microbial compounds have been reported to show inhibitory activity against *Pf*MQO.

This study aimed to screen and identify microbe-derived compounds that inhibit *Pf*MQO, and to assess its characteristics as antimalarial agents. We subjected extracts of Indonesia-originated fungi and actinomycetes deposited in the Biotech Center-BPPT Microbial Culture Collection (BioMCC) of the National Research and Innovation Agency (BRIN) to *Pf*MQO enzymatic reaction and screened them to obtain and identify the enzyme inhibitors using a high throughput screening (HTS) system. We also tested the inhibitory activity of the identified bioactive compound against the proliferation of the *P. falciparum* 3D7 cell in vitro, as well as its toxicity against several mammalian cells in vitro to confirm its specificity.

## 2. Results

### 2.1. HTS to Obtain Active Microbial Extract against PfMQO

**Figure 1:**
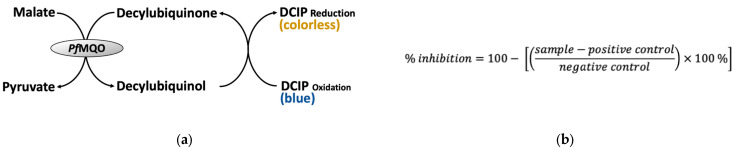
Principle of *Pf*MQO enzyme-based assay. (**a**) *Pf*MQO assay reaction. Reduction in DCIP_ox_ to DCIP_red_ will turn the reaction color from blue into colorless, so the reaction can be simply monitored by measuring the absorbance of the reaction mixture at a wavelength of 600 nm. (**b**) Formula for calculating the *Pf*MQO inhibitory activity. Sample, difference of A_660nm_ of reaction mixture containing the sample measured at 20 min and 0 min of reaction; positive control, average difference of A_600nm_ of reaction mixture without the addition of the substrate measured at 20 min and 0 min of reaction; negative control, average difference of A_600nm_ of reaction mixture without the addition of sample measured at 20 min and 0 min of reaction.

Primary screening was carried out by subjecting 1600 microbial broth extracts, composed from 800 fungi and 800 actinomycetes extracts, into the *Pf*MQO assay system (Figure 1) on a 96-well plate format. 

**Figure 2:**
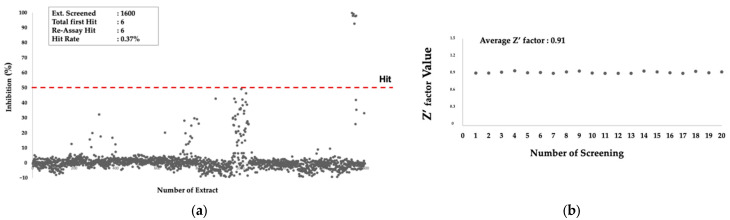
Primary screening of microbial extracts against *Pf*MQO. (**a**) Inhibitory activity of 1600 microbial extracts against *Pf*MQO; red-dashed line represents threshold line; (**b**) z’factor value of each assay batch.

The extracts that showed inhibitory activity more than 50% were regarded as primary hits. There were six extracts identified as primary hits, representing an overall hit rate of 0.37% (Figure 2a). The average of the z’factor value, a statistical parameter for monitoring the quality of the data from HTS, was 0.91 (Figure 2b), which is regarded as an excellent performance. All of the primary hits were produced by fungi. Since the assay system monitored the *Pf*MQO enzymatic reaction by a kinetic mode (time-course monitoring), we also checked whether the hits showed false positive results by observing the reaction color. The color of all hit-containing reactions remained blue, indicating that the hits were the true hits.

**Figure 3:**
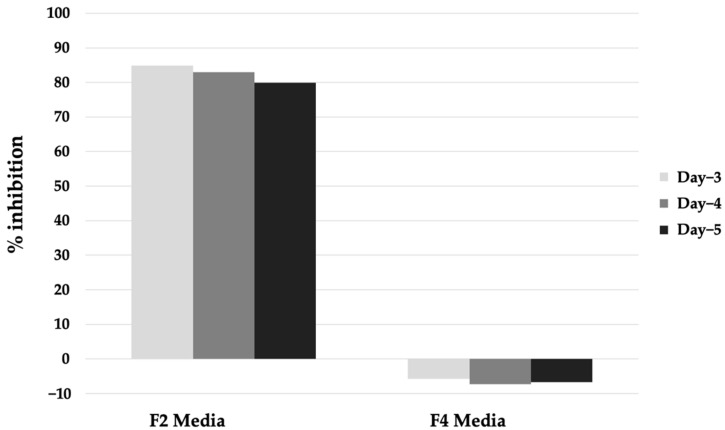
Inhibitory activity of extract of Aspergillus sp. BioMCC f.T.8501 cultured in F2 and F4 medium against PfMQO.

The re-assay of these hits against *Pf*MQO using the same samples resulted in a similar result. Thus, we then revived the microbes that produced these hits from the cryopreserved stock and tested the inhibitory activity of the extracts against *Pf*MQO. Surprisingly, only one of them was successfully revived. The inhibitory activity of the extract remained similar to that of the first screening result when the microbe was cultured in medium F2, but not in medium F4 (Figure 3).

The active extract was produced from the culture broth of *Aspergillus* sp. BioMCC f.T.8501, as identified based on its morphological characterization carried out by macroscopic and microscopic observation (Supplementary Data Figures S1 and S2). The strain formed a yellowish colony on the MEA medium and the conidiophore was varied in length and smooth. 

### 2.2. Preliminary Extraction Test

**Figure 4:**
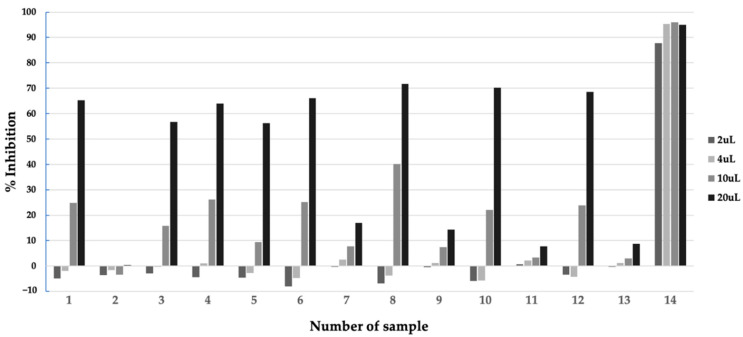
Inhibitory activity of samples from preliminary extraction test of extract of *Aspergillus* sp. BioMCC f.T.8501 culture broth in F2 medium against *Pf*MQO. Sample description: **1**, Supernatant; **2**, Supernatant (1/4 volume); **3**, Supernatant, pH 2, incubated at 60 °C for 1 h; **4**, Supernatant, pH 7, incubated at 60 °C for 1 h; **5**, Supernatant, pH 9, incubated at 60 °C for 1 h; **6**, Supernatant, pH 2, extracted with ethyl acetate, solvent layer; **7**, Supernatant, pH 2, extracted with ethyl acetate, water layer; **8**, Supernatant, pH 2, extracted with butanol, solvent layer; **9**, Supernatant, pH 2, extracted with butanol, water layer; **10**, Supernatant, pH 8, extracted with ethyl acetate, solvent layer; **11**, Supernatant, pH 8, extracted with ethyl acetate, water layer; **12**, Supernatant, pH 8, extracted with butanol, solvent layer; **13**, Supernatant, pH 8, extracted with butanol, water layer; **14**, Mycelium, extracted with methanol.

Before extraction and isolation of the bioactive compounds from the active microbial extract, it is important to characterize the properties of the active compounds. We performed a preliminary extraction test to assess the stability of the compounds and suitable solvents for extracting the compounds, as well as the localization of the active compounds in the culture broth, using a small amount of the extract, as described in the Materials and Methods. The test revealed that the mycelium extracted by methanol showed the highest inhibitory activity, compared to that of the supernatant fraction (Figure 4). We then extracted the remaining mycelium fraction of the extract by methanol and obtained 2.9 g of the dried extract.

### 2.3. Purification and Identification of Bioactive Compounds

**Figure 5:**
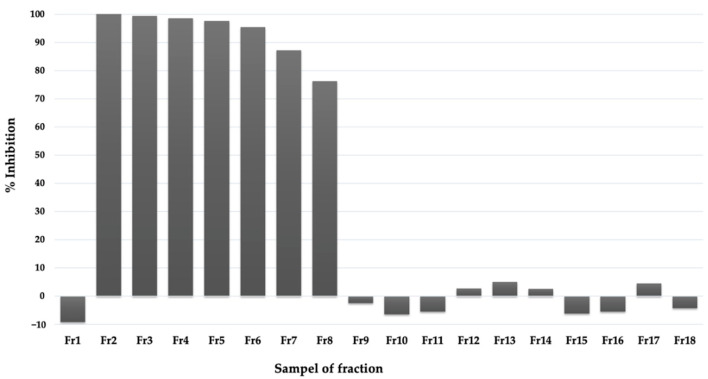
Inhibitory activity of fractions of active extracts eluted by mixed chloroform-methanol solvent from silica gel column chromatography against *Pf*MQO (see text for detail).

The methanol extract was then subjected to a silica open column and eluted by chloroform: methanol mixed solvent with various ratios (see Section 4). Each fraction eluted from the column was subjected to the *Pf*MQO assay. Early fractions (Fr2-Fr8) showed high inhibitory activity, which indicated that the active compounds might be relatively nonpolar (Figure 5). We then analyzed these active fractions with silica TLC with 100% chloroform and visualized them under UV at a wavelength of 254 nm. Fr2 and Fr3 showed similar patterns with a bold spot. The other fractions showed different patterns compared to those of the first two fractions (see Supplementary Data Figure S3). Based on their activities and pattern similarity and the spot boldness of the TLC result, we decided to mix Fr2 and Fr3 (and named as FrA accordingly) and subjected them for further purification process.

**Figure 6:**
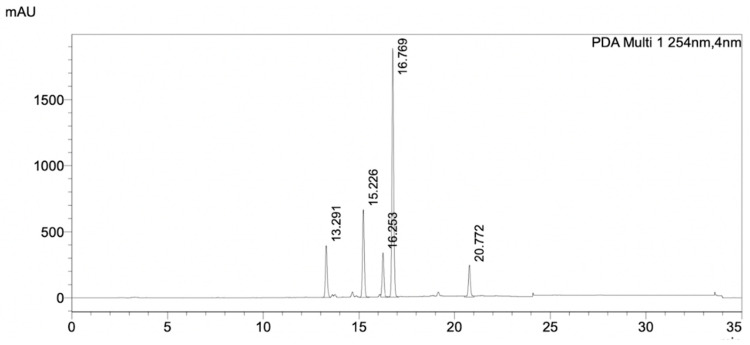
HPLC chromatogram of FrA from silica gel chromatography, separated by a C_18_ column. The data was taken by PDA detector. Detail of the HPLC condition is described in Section 4. The chromatogram showed the UV absorbance profile of the sample at wavelength of 254 nm.

**Figure 7:**
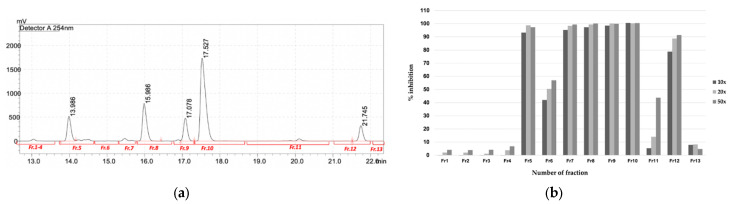
Purification of active compounds from FrA. (**a**) Semi-preparative HPLC chromatogram of the mixed active fraction, separated by a C_18_ column. The chromatogram showed UV absorbance profile of the sample at wavelength of 254 nm. Red bars underneath the chromatogram represent time-coursed sampling of fractions collected during the analysis. (**b**) Inhibitory activity of fractions collected from semi-preparative HPLC against *Pf*MQO. Sample was concentrated 10× (dark bar), 20× (light bar) or 50× (mid-dark bar) to represent a dose response of each sample.

The HPLC profile of FrA showed five major peaks with high intensities and well-separated (Figure 6). Each peak, including the non-peak fraction, was isolated by semi-preparative HPLC (Figure 7a) and subjected to the *Pf*MQO assay. All of these peaks showed an inhibitory activity against *Pf*MQO (Figure 7b). Due to the limited amount, we chose Fr10 (corresponded to the 4th major peak with r.t. of 17.527 s) to be further characterized.

**Figure 8:**
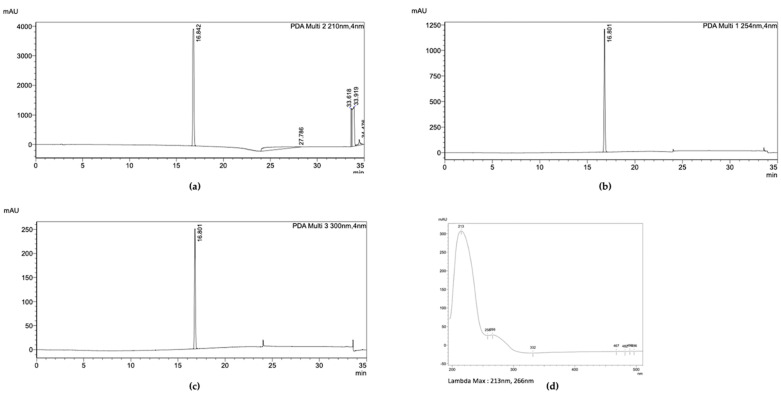
HPLC analysis of Fr10. The chromatogram showed the UV absorbance profile of Fr10 at wavelength of (**a**) 210 nm, (**b**) 254 nm, and (**c**) 300 nm. (**d**) UV spectrum of Fr10.

**Figure 9:**
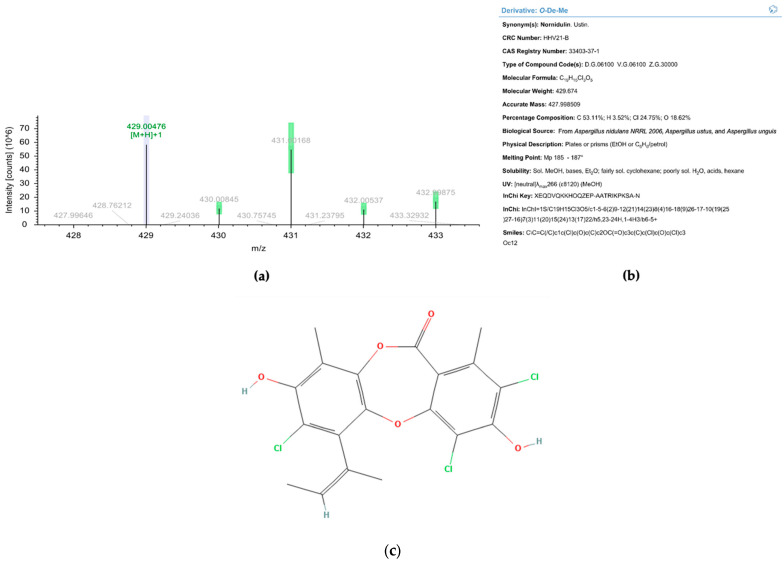
Liquid chromatography-mass spectrometry analysis of Fr10. (**a**) Electropherogram of Fr10 taken at positive mode. (**b**) Identification result of Fr10 from Dictionary of Natural Product database, based on UV spectrum and MS data. (**c**) Molecular structure of nornidulin (Pubchem ID 20056625).

When Fr10 was subjected for HPLC profiling, a single peak appeared on the HPLC chromatograms taken at 210 nm, 254 nm, and 300 nm (Figure 8a–c), suggesting that the Fr10 composed from a single compound. The LC-MS analysis results of this fraction revealed that the molecular weight of the compound was 429.00476 g/mol, with a molecular formula of C_19_H_15_Cl_3_O_5_ (Figure 9). We then searched the identity of the compound based on the LC-MS and UV spectrum (Figure 8d) data in the Dictionary of Natural Product database and identified the compound as nornidulin.

### 2.4. Characterization of the Purified Compound

**Figure 10:**
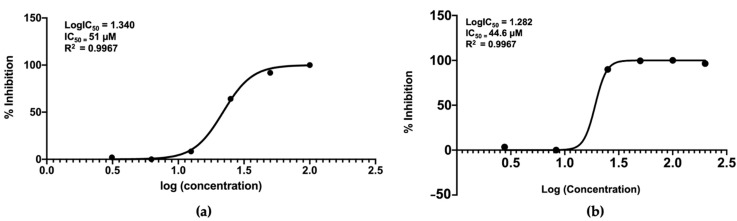
Inhibitory activity of nornidulin against *Pf*MQO (**a**) and proliferation of *P. falciparum* 3D7 cell in vitro, using *p*LDH assay method (**b**). The graph was drawn using GraphPad Prism V8 software.

**Figure 11:**
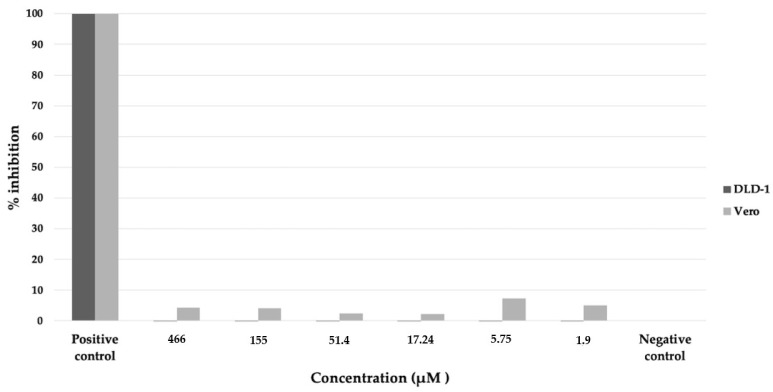
Cytotoxicity assay of nornidulin against colorectal adenocarcinoma (DLD-1, dark bar) and African green monkey kidney (Vero, light bar) cell lines. The assay was performed using CCK-8 method (see Materials and Methods). Positive control, culture medium without cell; negative control, cell culture without nornidulin.

The nornidulin inhibited *Pf*MQO and the proliferation of *P. falciparum* 3D7 in vitro at IC_50_ of 51 µM and 44.6 µM, respectively (Figure 10). No toxicity to mammalian cell cultures was observed when the nornidulin was tested against the mammalian cell cultures (colorectal adenocarcinoma (DLD-1) and the African green monkey kidney (Vero) cell lines) up to 466 µM (Figure 11), resulting in a selectivity index of more than 10. However, we observed a hemolysis during *p*LDH assay at a concentration of 466 µM.

## 3. Discussion

This study aimed to assess the potential of microbial resources for the discovery of the inhibitor of *Pf*MQO, an attractive target for anti-malarial drug discovery. We subjected 1600 microbial extracts into HTS against *Pf*MQO and obtained 6 extracts that were inhibited *Pf*MQO. Surprisingly, all of them were produced from fungi. This was consistent with the previous report, where none of the actinomycete’s extract (which employed more than 7700 extracts) showed inhibitory activity against *Pf*MQO, as well as *Pf*DHODH, another validated target involved in *P. falciparum* mtETC [12]. The *Pf*DHODH inhibitors that have been found are altenusin, mitorubrinol, and mitorubrinic acid, which were isolated from the Indonesian fungus *Talaromyces pinophilus* BioMCC f.T.3979 [15]. Therefore, fungi could become a useful source for anti-malarial drug discovery, particularly for searching the inhibitors of targets in plasmodial mtETC. At the same time, through this study, we also demonstrated the usefulness of Indonesian microbial strains as a source for searching inhibitors of *Pf*MQO.

In this study, we identified nornidulin, produced by *Aspergillus* sp. BioMCC f.T.8501, as an inhibitor of *Pf*MQO. Nornidulin is a depsidone derivative that is produced by marine- and soil-derived fungi, isolated first in 1945 from *Aspergillus nidulans*, and later from *Aspergillus ustus* and *Aspergillus unguis* [16,17,18]. *Aspergillus unguis* is known as a depsidones-producing fungus, which accounted for approximately 30% of the total produced compounds, along with depsides, phthalides, cyclopeptides, indanones, diaryl ethers, pyrones, benzoic acid derivatives, orcinol/orsenillate derivatives, and sesterpenoids. Nornidulin has been reported to show bioactivity as a larvicide and an antimicrobial. It is also reported to show bioactivity against cancer cell lines, animal growth promotion, antimalaria, and antioxidant [19]. Nornidulin inhibited aromatase with a IC_50_ value of 4.6 µM and showed radical scavenging activity in the xanthine/xanthine oxidase assay [20].

The bioactivity of nornidulin as an antimalarial agent was reported previously; it was demonstrated to inhibit the proliferation of the *P. falciparum* K1 strain at IC_50_ > 23.27 µM [14]. In this study, we demonstrated that nornidulin also inhibited the proliferation of the *P. falciparum* 3D7 strain at IC_50_ of 44.6 µM, and inhibited *Pf*MQO at IC_50_ of 51 µM. We did not further investigate the inhibitory mechanism of nornidulin against the proliferation of *P. falciparum* cell, but we found that the extract of *Aspergillus* sp. BioMCC f.T.8501 culture broth did not inhibit the *Pf*DHODH enzymatic reaction (data not shown). Therefore, we assumed that nornidulin inhibited the proliferation of *P. falciparum* cell by inhibiting *Pf*MQO specifically.

We also assessed the cytotoxicity of nornidulin against the DLD-1 and Vero cell lines. The compound did not affect the proliferation of the two cell lines at the highest concentration (466 µM), resulting in a selectivity index of more than 10. However, we observed hemolysis of RBC used in *p*LDH assay at this concentration (data not shown), indicating that RBC might be more sensitive to nornidulin compared to the tested mammalian cell lines.

In a previous study, systematic investigation was carried out to assess the cytotoxicity of phenolic polyketides from *Aspergillus unguis* against six cancer cell lines. Most of the isolated compounds showed cytotoxicity against all tested cell lines, with IC_50_ values ranging from 2.5 to 46.9 µM [21]. Another study isolated and characterized four new diphenyl ethers, Aspergillus ether GJ, and a new depsidone, emeguisin D, together with 18 known compounds from the endophyte *Aspergillus unguis* BCC54176. All of these components showed antibacterial activity, but none of these compounds were cytotoxic to cancer cells (MCF-7 and NCI-H187) and non-cancer cells (Vero) [14]. Since we did not observe cytotoxicity of nornidulin, we learned that not all depsidone derivatives exhibited cytotoxicity against mammalian cells.

## 4. Materials and Methods

### 4.1. Ethical Approval

This study was approved by the administration of the Health Research Ethics Committee (HREC), Faculty of Medicine, Universitas Brawijaya, Malang, East Java, Indonesia.

### 4.2. Microbial Extract Preparation

A total of 1600 microbial extracts, composed from 800 actinomycetes and 800 fungi extracts each, were prepared using an Indonesia-originated microbial strain collection deposited at the Biotech Center-BPPT Microbial Culture Collection of the National Research and Innovation Agency (BRIN). All microbes were revived from cryopreserved stocks on a malt extract agar (MEA) medium (2% malt extract, 2% glucose, 0.1% peptone, and 2% agar) for fungi, or International Streptomyces Project 4 (ISP 4) medium (1% soluble starch, 0.1% MgSO_4_.7H2O, 0.1% NaCl, 0.2% (NH_4_)_2_SO_4_, 0.25% CaCO_3_, and 2% agar) for actinomycetes. Actinomycetes were cultured in 2 types of media: C medium (2% rice powder, 1% glucose, 2% soybean meal, 0.5% yeast extract, 0.25% NaCl, 0.32% CaCO_3_, 0.2% mineral solution, pH 7.4) and A21 medium (0.5% glucose, 0.2% tryptone, 0.4% CaCO_3_, 0.2% NaCl, 0.05% KH_2_PO_4_, pH 7.0). Fungi were cultured in 4 types of media: F medium (2% rice powder, 1% glucose, 2% soybean meal, 0.1% KH_2_PO4, 0.05% MgSO_4_.7H_2_O), F15 medium (3% glucose, 2% glycerol, 1% dextrin, 1% malt extract, 2% yeast extract, 0.1% tryptone, 0.1% NH_4_NO_4_, 0.1% KH_2_PO_4_, pH 6.5), F2 medium (2% malt extract, 1.1% glucose, 0.22% yeast extract, 0.05% K_2_HPO_4_, 0.01% MgSO_4_.7H_2_O, 0.001% FeCl_4_, 0.000178% ZnSO_4_, 0.00055% CaCl), and F4 medium (0.5% malt extract, 1% glucose, 4% dextrin, 0.05% K_2_HPO_4_, 0.5% polypeptone, 0.5% soybean meal, 0.2% yeast extract, pH 6.0). Each strain was cultured in 50 mL medium in a 250 mL Erlenmeyer flask and shaken in a rotary shaker at 220 rpm and 28 °C for 7 days. The microbial culture broth was extracted by an equal volume of butanol and dried up in a vacuum centrifugal concentrator. The dried extract was dissolved in dimethyl sulfoxide (DMSO), so the extract was concentrated 25 times compared to its initial volume.

### 4.3. PfMQO Assay

*Pf*MQO enzyme was prepared using *Pf*MQO-expressing recombinant *Escherichia coli,* as described by Hartuti et al. (2018) [11]. The principle of the *Pf*MQO assay is shown in Figure 2a. An assay mix solution was prepared from 50 mM HEPES-KOH (pH 7.5), 1 mM KCN, 60 µM decylubiquinone, 120 µM DCIP (blue), and 3 µg of *Pf*MQO-membrane fraction. Further, 193 µL of the assay mix was transferred to a 96-well microplate, and 2 µL of microbial extract was added. The reaction was started by the addition of 5 µL of 400 mM sodium-*L*-malate (Wako) and subsequently mixed using a plate mixer (800–1000 rpm) for 30 seconds. The absorbance of the mixture was recorded by a SpectraMax^®®^ Paradigm^®®^ multi-mode multiplate reader (Molecular Devices, California, USA). The inhibitory activity was calculated using a formula, as described in Figure 2b. The reaction mixture without the addition of the substrate and microbial extract was regarded as the positive control (PC) and negative control (NC), respectively.

The performance of the screening system was evaluated by calculating the statistical parameter, z’factor, with the following Equation [22]

$$z’\mathrm{factor}=1-\frac{3\mathrm{SD}\;\mathrm{of}\;\mathrm{PC}\;+3\mathrm{SD}\;\mathrm{of}\;\mathrm{NC}}{\left|\mathrm{mean}\;\mathrm{of}\;\mathrm{PC}\;-\;\mathrm{mean}\;\mathrm{of}\;\mathrm{NC}\right|}$$

where SD is the standard deviation; PC is the positive control; and NC is the negative control.

### 4.4. Lactate Dehydrogenase (pLDH) Assay

**Figure 12:**
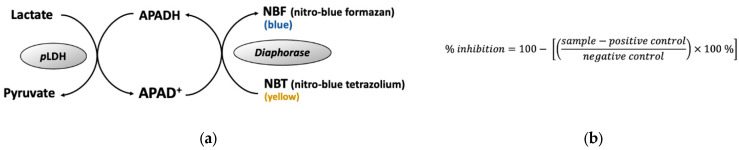
Principle of *p*LDH assay. (**a**) *p*LDH assay reaction, (**b**) formula for calculating the inhibitory activity of the proliferation of *P. falciparum* cell. Sample, A_650nm_, of reaction mixture with the presence of sample; positive control, A_650nm_, of reaction mixture with the presence of 1 µM atovaquone; negative control, A_650nm_, of reaction mixture with the presence of 0.4% DMSO.

The principle of *p*LDH assay is shown in Figure 12a. Red blood cell (RBC) type O+ was obtained from the local Red Cross. *P. falciparum* 3D7 was maintained in a RPMI-1640 medium (supplemented with Albumax II (Gibco-Thermo Fisher Scientific, Waltham, MA, USA), and contained RBC (3% hematocrit). *P. falciparum* cell was synchronized into the ring form stage using 5% sorbitol, and then adjusted so the parasitemia was 0.3%. The sample was added to 96-well microplate, followed by the addition of 100 μL of the *P. falciparum* cell culture. The cell was incubated for 3 days at 37 °C, 5% CO_2_, 5% O_2_. Cold PBS was added to the cell culture, as much as 200 μL, then centrifuged at 1700× *g* for 10 min. After discarding the supernatant, 100 µL of LDH reaction mixture (100 mM Tris-HCl pH 8.0, 50 mM sodium-*L*-lactate, 0.25% (*v*/*v*) Triton X-100), 0.2% nitro blue tetrazolium (NBT), 50 µg/mL 3-acetyl pyridine NAD (APAD), and 0.05 U diaphorase) was added into each well and incubated at 37 °C for 30 min before the absorbance was measured at 650 nm using a microplate reader. Inhibition activity was calculated using formula in Figure 12b. Cell cultures with the addition of DMSO (final concentration 0.4%) and atovaquone (final concentration 1 µM) were regarded as negative and positive controls, respectively.

### 4.5. Preliminary Extraction Test

A small part (1–5 mL) of microbial culture broth was centrifuged to separate the mycelium from the supernatant. The mycelium was extracted by methanol. A part of the supernatant was extracted by butanol or ethyl acetate under different pH levels (2 and 8), then centrifuged at 10,000× *g* for 10 min to separate the solvent and water layer. The other part of the supernatant was heated at 60 °C for 1 h under pH 2, 7 or 9, then centrifuged at 10,000× *g* for 10 min. All samples were subjected to the *Pf*MQO assay.

### 4.6. Thin Layer Chromatography (TLC)

TLC was carried out on a silica TLC plate (Merck, Darmstadt, Germany) in a glass chamber containing organic solvent (as described in the text). The spot on the TLC plate was visualized under UV light or by spraying phosphomolybdic acid.

### 4.7. Open Column Chromatography (OCC)

The dried extract was mixed with silica gel 60 F_254_ resin (0.063–0.040 mm, Merck, Darmstadt, Germany), then loaded into a glass column (4 cm diameter, 40 cm length). Compounds from the extract were eluted by a mixed solvent of chloroform (CHCl_3_) and methanol (CH_3_OH) step wisely (100% CHCl_3_, 90% CHCl_3_: 10% CH_3_OH, 80% CHCl_3_: 20% CH_3_OH, 50% CHCl_3_: 50% CH_3_OH, 100% CH_3_OH), and the eluted solvent was collected using a fraction collector. A small part of these fraction was dried up in a vacuum concentrator, dissolved in DMSO, and then subjected to the *Pf*MQO assay.

### 4.8. Analytical and Semi-Preparative HPLC

Analytical HPLC was performed using the Prominence modular HPLC system equipped with an SPD-M30A photodiode array detector and an LC-20A dual channel solvent delivery pump (Shimadzu, Kyoto, Japan). In total. 10 µL of the sample was separated in a C_18_ Sunfire^®^ OBD Column 100A (2.5 µm particle size, 10 mm diameter, 250 mm length, Waters, MI, USA), and eluted with a mixed water—acetonitrile (A—B) solvent (containing 0.05% trifluoroacetic acid) at a flow rate of 1 mL/min under the conditions below: flow 40% A for the first 20 min, increase to 100% A for the next 10 min, then decrease to 40% A for 5 min.

Semi-preparative HPLC was performed using the Prominence modular HPLC system equipped with a SPD-20A UV detector and an LC-20A dual channel solvent delivery pump (Shimadzu, Kyoto, Japan). In total, 100 µL of the sample was injected into a C_18_ Sunfire® OBD Prep column 100A (5 µm particle size, 10 mm diameter, 250 mm length, Waters, MI, USA), and eluted with mixed water—acetonitrile (A—B) solvent (containing 0.05% trifluoroacetic acid) at a flow rate of 4 mL/min for 55 min under the conditions below: flow 40% A for the first 20 min, increase to 100% A for the next 30 min, then decrease to 40% A for 5 min.

### 4.9. LC-MS Analysis

LC-MS analysis was performed using a HPLC system (Dionex Ultimate 3000 RSC, Thermo Fisher Scientific, MA, USA) connected with a high-resolution mass spectrometer Q-Exactive (Thermo Fisher Scientific, MA, USA). In total, 100 µL of the sample was injected into the HPLC equipped with Hypersil GOLD aQ C_18_ polar-endcapped HPLC column (50 mm length, 1 mm diameter, 1.9 µl particle size, Thermo Fisher Scientific, MA, USA) and eluted with a mixed water—acetonitrile (A—B) solvent (both containing 0.1% formic acid at flow rate 40 µL/min for 30 min) under the conditions below: flow 5% B for the first 2 min, then increase to 60% B for 13 min, further increase to 95% B for the next 7 min before keeping at this concentration for 3 min, then decrease and keep at 5% B for 5 min. The sample was detected by MS (full scan at 70.000 resolution) and MS^2^ (data dependent at 17,500 resolutions). Data were processed with compound discoverer software using mzCloud MS/MS library version 3.2.

### 4.10. Cytotoxicity of Bioactive Compound on Mammalian Cells

Human colorectal adenocarcinoma cells (DLD-1) and the kidney of an African green monkey cell derived (Vero) were cultured in Dulbecco’s Modified Eagle Medium (DMEM, Gibco-Thermo Fisher Scientific, MA, USA) supplemented with 10% of inactivated fetal bovine serum (Gibco-Thermo Fisher Scientific, MA, USA). The cells were transferred to a 96-well microplate, so the initial cell number was 1.25 × 10^4^ cells 5 × 10^3^ cells for DLD-1 and Vero, respectively, per well, then incubated at 37 °C, 5% CO_2_ for 24 h. The sample was added as much as 0.4 µL or a volume so the final concentration of DMSO in the culture was less than 1%, then incubated at 37 °C, 5% CO_2_ for 48 h. After being washed with 100 µL of PBS, the cell was resuspended with 100 µL of DMEM containing 10% of Cell Counting Kit-8 (Dojindo, Kumamoto, Japan) and placed in the incubator at 37 °C, 5% CO_2_ for 3 h. The absorbance of each well was measured at 450 nm by a plate reader (Spectramax Paradigm, Molecular Devices, San Jose, CA, USA).

## 5. Conclusions

In this study, we reported nornidulin as microbe-derived inhibitor of *Pf*MQO for the first time. We also demonstrated that nornidulin inhibited the proliferation of *P. falciparum* 3D7 strains, but not DLD-1 and Vero cell lines with a selectivity index of more than 10. Since we isolated nornidulin from an Indonesia-originated soil fungus, *Aspergillus* sp. BioMCC f.T.8501, we also emphasized the potential of Indonesian microbial strains as a promising source for anti-malarial drug discovery.

## Data Availability

Data is contained within the article and supplementary material.

## Supplementary Materials

The following supporting information can be downloaded at: https://www.mdpi.com/article/10.3390/ph16020268/s1, Figure S1: Morphology of *Aspergillus* sp. BioMCC f.T.8501, macroscopic form of the fungus on MEA medium; Figure S2: Morphology of *Aspergillus* sp. BioMCC f.T.8501, Microscopic shape the conidiophore; Figure S3: Thin layer chromatography of active fractions (Fr2-Fr8) from silica gel column chromatography.
