# Supplementary material for: Nornidulin, A New Inhibitor of Plasmodium falciparum Malate: Quinone Oxidoreductase (PfMQO) from Indonesian Aspergillus sp. BioMCC f.T.8501
Source: Pharmaceuticals (Basel). 2023 Feb 10;16(2):268. doi: 10.3390/ph16020268 (PMC9964459; doi:10.3390/ph16020268)
Supplement: Supplementary file 1 [file pharmaceuticals-16-00268-s001.zip › pharmaceuticals-2177421-supplementary.pdf]

Supplementary Data

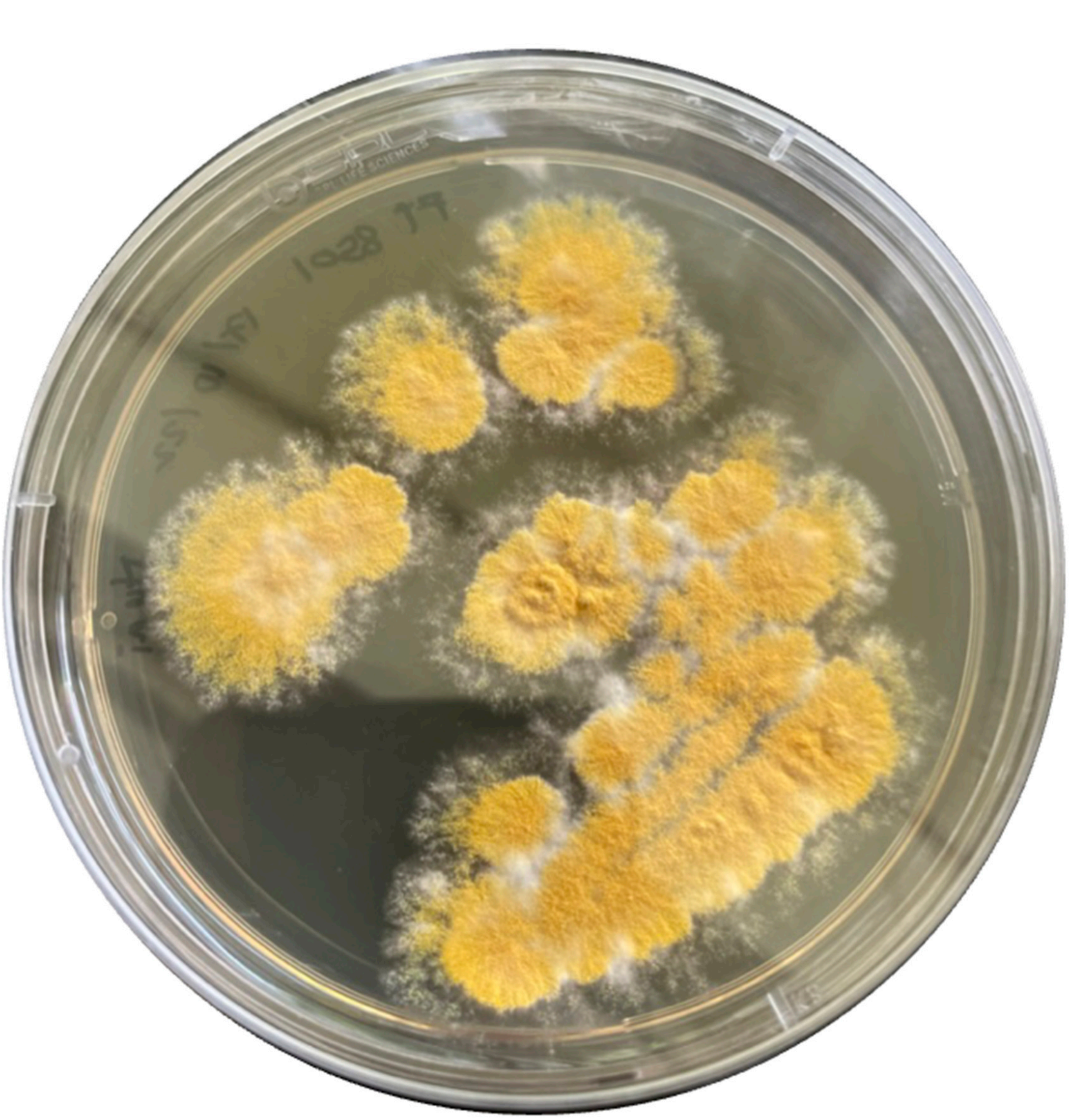

Figure S1. Morphology of *Aspergillus* sp. BioMCC f.T.8501, macroscopic form of the fungus on MEA medium.

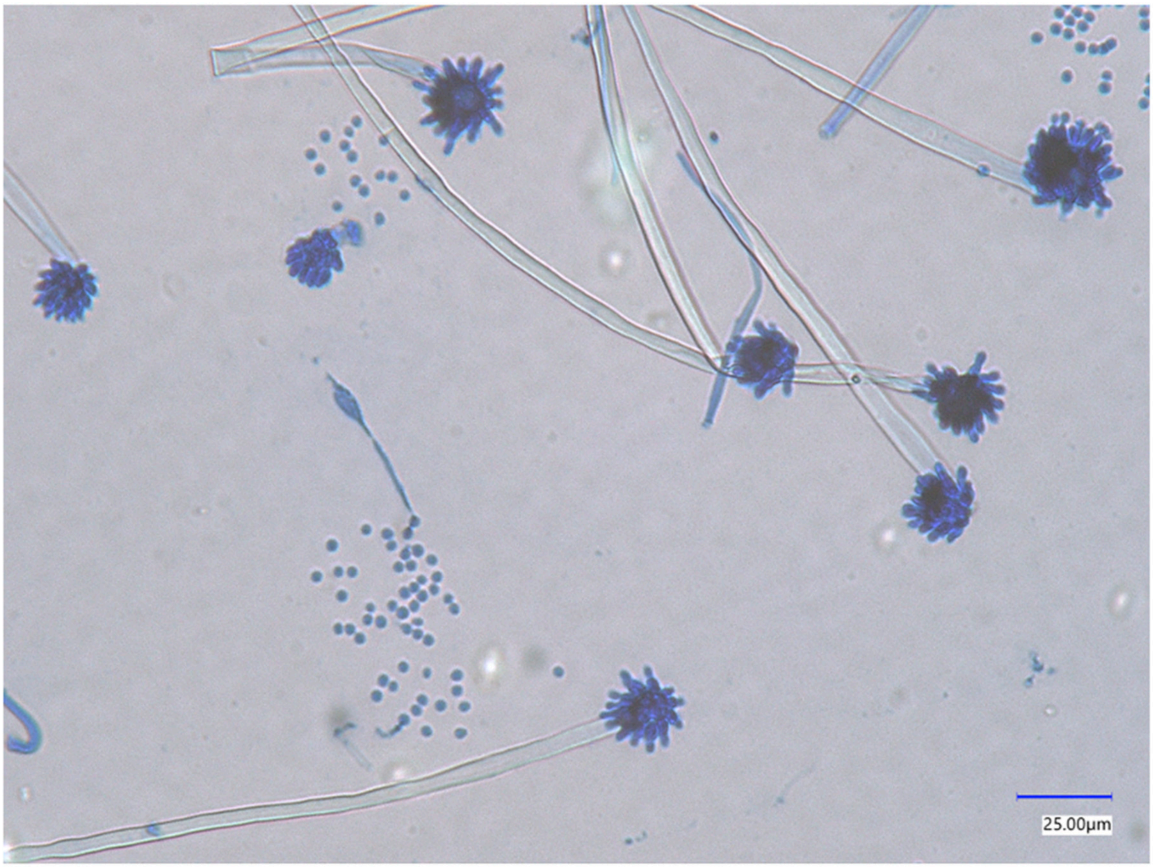

Figure S2. Morphology of *Aspergillus* sp. BioMCC f.T.8501, Microscopic shape of the conidiophore

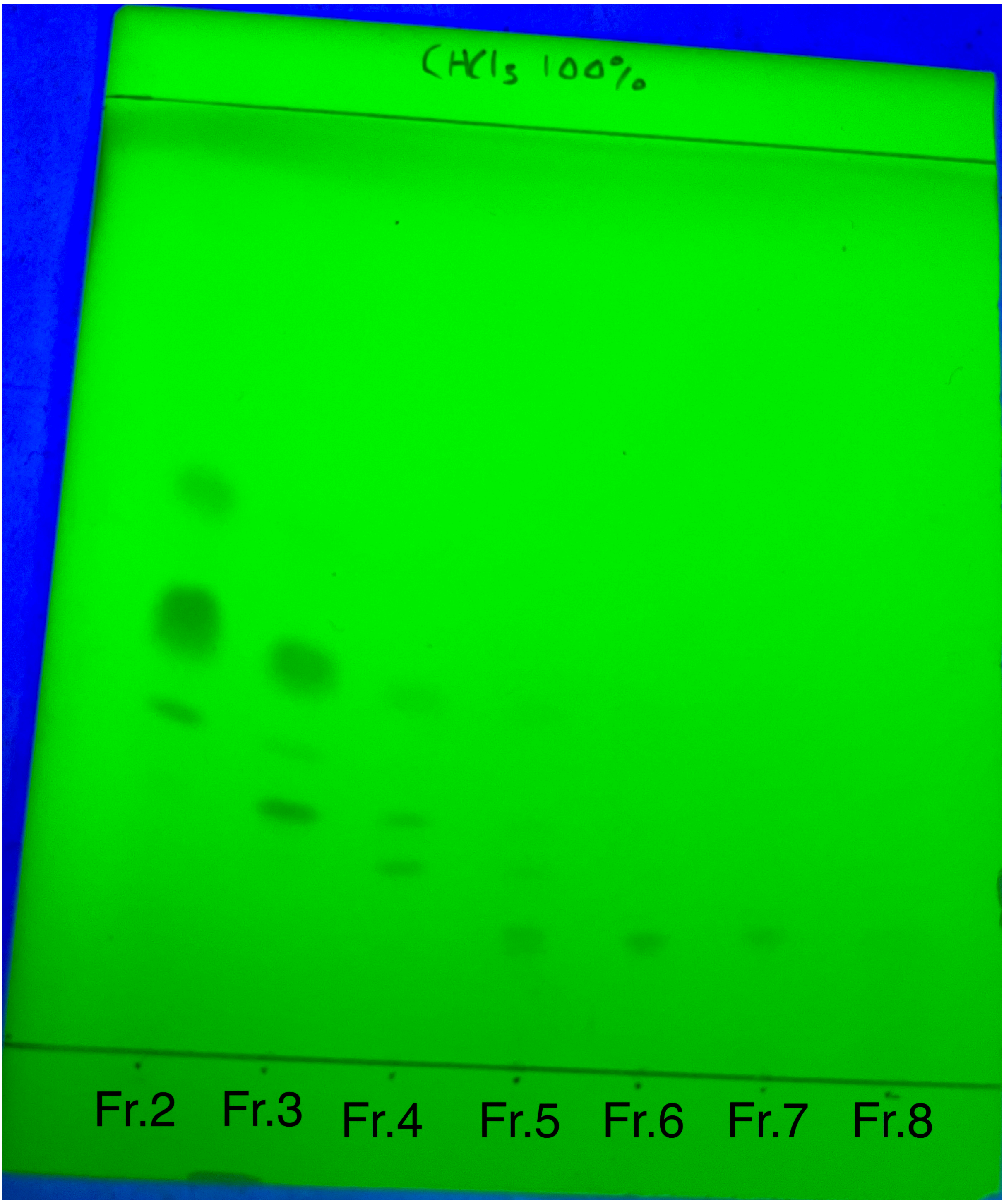

Figure S3. Thin layer chromatography (TLC) of active fractions (Fr2-Fr8) from silica gel column chromatography. TLC was developed with 100% chloroform and visualized under UV at wavelength of 254 nm.
